# A Physics-Informed Dual-Branch LSTM Network for UAV Position and Attitude Estimation

**DOI:** 10.3390/s26134287

**Published:** 2026-07-06

**Authors:** Weizheng Liang, Siqi Meng, Ruicheng Zhang, Qianda Luo

**Affiliations:** College of Electrical Engineering, North China University of Science and Technology, Tangshan 063210, China; liangwh@ncst.edu.cn (W.L.); sqmeng@stu.ncst.edu.cn (S.M.); luoqianda@ncst.edu.cn (Q.L.)

**Keywords:** UAV position and attitude estimation, inertial navigation, physics-informed learning, inertial odometry, long short-term memory (LSTM), digital twin, precision agriculture

## Abstract

To mitigate error accumulation and long-term drift in unmanned aerial vehicle (UAV) position and attitude estimation using purely inertial measurement unit (IMU) data, this paper presents a dual-branch physics-informed long short-term memory (DPI-LSTM) network incorporating shared temporal encoding, a dual-branch structured regression framework, and physical consistency constraints. The model employs a long short-term memory (LSTM)-based temporal encoder to extract temporal features from IMU time-window sequences. Established inertial kinematic relationships are embedded into the dual-branch LSTM framework as loss constraints, providing physics-based regularisation to guide the network during training. By modelling translational and rotational states separately through the position and attitude branches, the model improves stability and physical interpretability while retaining the advantages of task decoupling. Systematic experiments were conducted on the University of Zurich First-Person View (UZH-FPV) Drone Racing dataset, and comparisons were made with traditional inertial navigation methods and representative deep learning-based inertial odometry approaches. The experimental results indicate that the proposed model demonstrates a measurable reduction in positional root mean square error (RMSE) on the evaluated test sequences, decreasing the RMSE to 0.0654 m, which represents a reduction of more than 20% when compared with inertial odometry network (IONet), convolutional neural network–long short-term memory (CNN–LSTM), and robust neural inertial navigation (RoNIN). Further ablation studies and cross-sequence evaluation indicate that the physical consistency constraints and the dual-branch architecture contribute to improved position estimation stability under the evaluated benchmark sequences. The proposed kinematically constrained framework provides a viable IMU-only position and attitude estimation module, laying the groundwork for future UAV digital twin and precision-agriculture applications where continuous and physically consistent position and attitude information is required.

## 1. Introduction

### 1.1. Background

With the continuous advancement of aviation technology and its deep integration with information technology (IT) [1,2,3,4], unmanned aerial vehicles (UAVs) have developed rapidly in recent years. Owing to their low cost and flexible deployment, UAVs have been widely applied worldwide, particularly in fields such as surveying, inspection and patrol, disaster search and rescue, and logistics [5,6,7]. As their application scope continues to expand, UAVs are increasingly influencing modern production and daily life. In agricultural scenarios, UAVs are increasingly used for growth-status assessment, crop monitoring, field mapping, and precision spraying [8,9,10], where stable low-altitude flight and accurate position and attitude estimation are essential for ensuring operational quality and safety.

The concept of the digital twin (DT) can be traced back to the “living model” developed by the National Aeronautics and Space Administration (NASA) during the Apollo missions [11,12], in which high-fidelity models were established on the ground to monitor, update, and predict the state and behaviour of spacecraft in orbit. The modern concept of the digital twin is generally attributed to Michael Grieves. A digital twin refers to a digital representation of a physical entity constructed in a virtual space, one which is continuously updated through real-time data to reflect the entity’s current state and support the prediction of its future evolution [13,14,15]. Structurally, a digital twin typically consists of three components: the physical entity, the digital model, and the data link that connects the two and enables information exchange.

An unmanned aerial vehicle digital twin (UAVDT) is not merely a framework for the visualisation and replay of historical flight data [16]; rather, it places greater emphasis on enabling real-time monitoring, short-term prediction, and risk assessment of the UAV’s position, attitude, and trajectory during flight, particularly under uncertainties such as complex manoeuvres and environmental disturbances. As a core component of a UAVDT, the digital model requires high accuracy and robustness to faithfully represent actual flight conditions and ensure engineering reliability. Precision agriculture refers to an agricultural management approach that uses sensing [17], positioning, data analysis, and automated equipment to support more accurate, efficient, and site-specific field operations. In this context, UAVs are widely used for low-altitude tasks such as crop inspection, field mapping, and precision spraying, where reliable position and attitude estimation is essential for operation monitoring, mission replay, trajectory assessment, and flight safety. Therefore, the development of stable position and attitude estimation algorithms provides a theoretical foundation for potential online monitoring and UAV digital twin applications in future agricultural scenarios.

### 1.2. Related Work

Currently, UAV position and attitude estimation and localisation methods can be broadly divided into two categories: filtering-based methods [18,19,20,21] and deep learning-based methods [21,22,23,24,25]. Among filtering-based methods, the Kalman filter (KF) and its variants are the most representative. Under reasonable assumptions regarding the system state-space model and the statistical characteristics of noise, these methods can provide minimum mean-square-error state estimation for variables such as position and attitude by combining system dynamics with sensor observations.

In inertial measurement unit (IMU)-based Kalman filtering methods, position and attitude estimation relies on the continuous integration of inertial measurements [21]. In the presence of sensor noise and bias, integration errors accumulate over time, leading to significant drift, particularly in position estimation under IMU-only conditions [18]. To improve robustness under disturbances, several studies have refined filtering strategies. For example, Candan et al. proposed an attitude estimation method based on robust Kalman filtering (RKF) [19], which suppresses the influence of external acceleration by adaptively adjusting the measurement noise covariance. However, such approaches are generally more effective in stabilising attitude estimation than in maintaining long-term position accuracy.

To mitigate the drift caused by pure inertial integration, many studies have fused IMU measurements with external sensors. Among these, global navigation satellite system (GNSS)/inertial measurement unit (IMU) integration is one of the most widely adopted solutions. The IMU provides high-frequency motion information, while GNSS supplies position observations with high absolute accuracy. Fernandes et al. proposed a hybrid positioning method based on lie group theory [20], which jointly estimates position, velocity, attitude, and IMU bias within an extended Kalman filter framework and fuses GNSS observations with IMU measurements through a loosely coupled structure, thereby significantly improving positioning accuracy and long-term navigation stability. However, such methods rely on external observations and are therefore not directly applicable to IMU-only position and attitude estimation scenarios.

In recent years, data-driven methods based on deep learning have emerged as an important research direction for position and attitude estimation and inertial localisation because of their strong nonlinear modelling capability [22]. Zeinali et al. proposed IMUNet [23], a convolutional neural network (CNN)-based localisation architecture incorporating MobileResNet modules that combine deep convolutions with residual connections and are designed to improve localisation accuracy while reducing computational complexity. Chen et al. proposed the inertial odometry network (IONet) [24], which formulates inertial localisation as a sequence learning problem and predicts displacement and heading changes directly from IMU time series using a two-layer bidirectional long short-term memory (LSTM) network. Similarly, robust neural inertial navigation (RoNIN) [25] utilises deep neural networks to learn motion representations from IMU data and directly regress motion states, thereby reducing error accumulation caused by noise and bias. RoNIN has been validated on datasets containing multiple devices and motion modes, demonstrating strong robustness and cross-scenario generalisation capability. Wu et al. proposed a CNN–LSTM-assisted GNSS tracking method [26], in which CNNs extract motion features from IMU data while LSTMs model temporal dependencies to estimate IMU bias and scale-factor errors, thereby suppressing position divergence during GNSS signal outages. In addition, hybrid methods combining neural networks with filtering frameworks have also been explored. For example, Liu et al. proposed a tight learned inertial odometry (TLIO) method [27], which predicts short-term three-dimensional displacements and their uncertainties from IMU sequences using a neural network and incorporates them as observations into an extended Kalman filter, thereby improving the stability of inertial odometry under IMU-only conditions.

More recently, physics-informed extensions of deep learning methods have been introduced to improve physical consistency in inertial localisation. Physics-informed neural networks (PINNs) incorporate system dynamics equations into the training process by embedding physical constraints into the loss function. For example, Sahoo et al. proposed the mobile robot pure inertial navigation-physics-informed neural networks (MoRPI-PINN) framework [28], which integrates two-dimensional inertial navigation equations into the loss function to achieve physically consistent mobile robot localisation using only IMU measurements.

Multi-task learning provides an effective strategy for sharing representations among related tasks [29]. Such architectures have also been successfully applied to pose-estimation problems in other autonomous systems. For instance, Park and D’Amico proposed the Spacecraft Pose Network v2 (SPNv2) for spacecraft pose estimation [30], where a shared feature encoder is coupled with multiple prediction heads for distinct objectives, including direct pose regression, keypoint prediction, and foreground segmentation. Within its pose-regression head, rotation and translation are treated as distinct yet coupled prediction targets—a design that provides a valuable reference for separately modelling rotational and translational components in UAV position and attitude estimation networks.

In summary, existing filtering-based inertial navigation methods are susceptible to noise, sensor bias, and drift accumulation under long-duration, purely inertial conditions, making it difficult to maintain stable position and attitude estimates. While existing deep learning-based inertial odometry methods predominantly focus on capturing temporal mapping relationships from raw IMU sequences, they seldom explicitly incorporate classical kinematic principles into their optimisation objectives. To address this limitation, this study embeds these established relationships as structured regularisation to guide the network’s training process. Furthermore, in complex low-altitude flight scenarios, GNSS sensors often struggle to balance positioning accuracy with signal availability, while the continuous operation of visual odometry imposes considerable computational and power consumption burdens. In precision agriculture, where UAVs often operate at low altitude for crop inspection and precision spraying, these limitations become even more critical, further motivating the need for position and attitude estimation methods with strong physical consistency constraints and stable estimation performance under sequence-level distribution shifts. At the same time, UAV digital twin systems require a continuous supply of stable, accurate, and physically consistent position and attitude information to support online monitoring, state assessment, and short-term trajectory prediction. Consequently, there is a need for a position and attitude estimation method that can simultaneously address temporal feature representation, physical consistency, and rotation–translation coupling.

### 1.3. Contributions

To address the aforementioned issues, this paper proposes a dual-branch physics-informed long short-term memory (DPI-LSTM) model for UAV position and attitude estimation using IMU data. The main contributions are as follows:

1. In addition to the data-fitting loss, this study incorporates constraints derived from established inertial kinematic relationships, including attitude propagation, coordinate transformation, and velocity–position integration consistency. By embedding these constraints into the training objective, the network is guided to learn position-and-attitude representations that align with explicit kinematic laws. This introduces physical interpretability into the otherwise black-box deep learning model by linking its position and attitude predictions to explicit inertial kinematic relationships, while also helping suppress drift and error accumulation.
2. Motivated by multi-task position and attitude estimation architectures that utilise shared feature representations alongside task-specific prediction heads for complementary state components such as rotation and translation, this work proposes a modelling framework comprising shared temporal encoding, dual-branch structured regression, and physical consistency constraints. This framework employs a shared LSTM encoder to generate a unified representation of windowed IMU sequences, from which position and attitude are regressed separately through two distinct output branches. The physical consistency constraints, derived from classical inertial kinematics, are enforced to maintain coherence between the rotational and translational state estimates, thereby enhancing tracking stability while preserving the benefits of task decoupling.
3. A systematic experimental framework was constructed based on the University of Zurich First-Person View (UZH-FPV) dataset, and the proposed model was comprehensively validated through comparisons with traditional inertial navigation approaches and learning-based inertial odometry approaches, as well as through ablation studies and cross-sequence evaluation. The experimental results demonstrate that DPI-LSTM exhibits consistent advantages in terms of position accuracy, estimation stability, and cross-sequence performance, thereby confirming the effectiveness of structured physical constraint modelling.

The remainder of the paper is organised as follows. Section 2 introduces the fundamental mathematical models involved in this work, providing a theoretical foundation for the subsequent sections. Section 3 presents temporal feature modelling based on LSTM. Section 4 formulates the physical consistency constraints adopted in this study. Section 5 describes the proposed dual-branch physics-informed Long Short-Term Memory (DPI-LSTM) model for UAV position and attitude estimation. Section 6 reports the experimental results and analysis, including comparative experiments, ablation studies, and cross-sequence evaluation. Finally, Section 7 summarises the main findings of this work and discusses future research directions.

## 2. Mathematical Model of UAVs

### 2.1. Definition of Coordinate Systems and Attitude Representation

**Figure 1:**
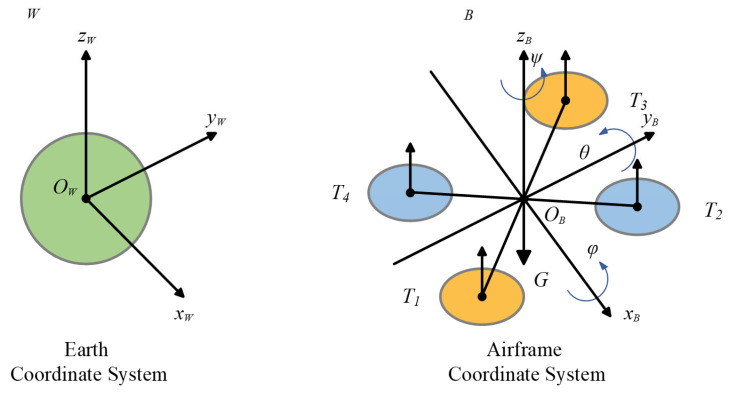
Schematic diagram of the coordinate systems.

In robotics and rigid-body modelling, the motion state of a body relative to a reference frame is commonly described using its translational position and rotational attitude. Following standard treatments in robot modelling and control, this study represents the UAV position in the world frame and its attitude using a unit quaternion [31]. A quadrotor UAV can be approximated as a homogeneous rigid body with an X-shaped configuration [18,32]. The origin of the body coordinate system B is defined at the IMU mounting position, and its axes are aligned with those of the IMU. The world coordinate system W follows a right-handed convention, in which the $z^{W}$-axis points vertically upward and gravity acts along the negative $z^{W}$-axis, as shown in Figure 1.

This study adopts a local, gravity-aligned world frame tailored for short-duration and low-altitude UAV position and attitude estimation. Within this local coordinate configuration, the Earth is assumed to be flat and non-rotating; thus, the effects of the Earth’s curvature, rotation, transport rate, and Coriolis acceleration are neglected, while gravity is treated as a constant vector within the world frame. Furthermore, the proposed learning-based estimator bypasses the complete initial alignment procedure typical of classical inertial navigation systems. Instead, the initial reference state is obtained directly from the dataset-provided trajectory, and explicit online gyroscope drift compensation is omitted prior to navigation.

The position and velocity of the UAV in the world frame W are denoted as follows:

(1) $$p^{W}=\left\{x^{W},y^{W},z^{W}\right\},\;v^{W}={\dot{p}}^{W},$$

Because Euler angles may suffer from gimbal lock under highly manoeuvrable conditions, leading to discontinuities in attitude representation, unit quaternions are adopted to represent attitude:

(2) $$q_{WB}={\left[q_{w},q_{x},q_{y},q_{z}\right]}^{T},\;\left\|q_{WB}\right\|=1,$$

the corresponding rotation matrix $R_{WB}\left(q\right)\in SO\left(3\right)$ satisfies the following coordinate transformation:

(3) $$r^{W}=R_{WB}\left(q\right)r^{B},$$

A common form of $R_{WB}\left(q\right)$ is given by the following:

(4) $$R_{WB}\left(q\right)=\left[\begin{array}{ccc} 1-2\left(q_{y}^{2}+q_{z}^{2}\right) & 2\left(q_{x}q_{y}-q_{w}q_{z}\right) & 2\left(q_{x}q_{y}+q_{w}q_{y}\right)\\ 2\left(q_{x}q_{y}+q_{w}q_{z}\right) & 1-2\left(q_{x}^{2}+q_{z}^{2}\right) & 2\left(q_{y}q_{z}-q_{w}q_{x}\right)\\ 2\left(q_{x}q_{y}-q_{w}q_{y}\right) & 2\left(q_{y}q_{z}+q_{w}q_{x}\right) & 1-2\left(q_{x}^{2}+q_{y}^{2}\right) \end{array}\right],$$

Furthermore, as the quaternions q and −q represent the same rotation, to ensure the continuity and smoothness of the attitude time series, sign continuity is adopted:

(5) $$\mathrm{if}\;q_{k}^{T}q_{k-1}<0,\;\mathrm{then}\;q_{k}=-q_{k},$$

### 2.2. IMU Measurement Model

To establish the physical link between inertial measurements and UAV motion states, the IMU measurements in the body frame B are divided into two parts: gyroscope angular velocity and accelerometer specific force, denoted by $\omega_{m}^{B}$ and $f_{m}^{B}$, respectively. Considering sensor bias and measurement noise, the IMU measurement model can be written as follows:

(6) $$\omega_{m}^{B}=\omega^{B}\left(t\right)+b_{g}\left(t\right)+n_{g}\left(t\right),$$

(7) $$f_{m}^{B}=f^{B}\left(t\right)+b_{a}\left(t\right)+n_{a}\left(t\right),$$

where $\omega_{m}^{B}$ denotes the gyroscope measurement, $f^{B}$ denotes the accelerometer measurement of specific force, $b_{g}$ and $b_{a}$ are the gyroscope and accelerometer biases, respectively, and $n_{g}$ and $n_{a}$ are zero-mean white noise terms. The bias terms are often approximated by random-walk processes:

(8) $${\dot{b}}_{g}\;=\;n_{bg},\;{\dot{b}}_{a}=n_{ba},$$

It should be noted that an accelerometer does not directly measure the linear acceleration in the world frame; instead, it measures the specific force relative to gravity. This relationship can be expressed as follows:

(9) $$f^{B}\left(t\right)=R_{BW}^{T}\left(q\right)\left(a^{W}\left(t\right)-g^{W}\right),$$

Accordingly, the world-frame acceleration can be obtained as follows:

(10) $$a^{W}\left(t\right)=R_{WB}\left(q\right)\left(f_{m}^{B}\left(t\right)-b_{a}\left(t\right)\right)+g^{W},$$

The above IMU measurement model provides the kinematic basis for constructing the physical consistency constraints used in this study. On the one hand, gyroscope angular velocity drives the temporal evolution of the quaternion. On the other hand, the measured specific force, after gravity compensation through attitude-based coordinate transformation, yields the world-frame acceleration $a^{W}$, which further participates in the integral consistency constraints for velocity and position. These constraints do not directly eliminate raw sensor noise or biases; rather, they enforce kinematic consistency across attitude propagation, coordinate transformation, and velocity–position integration during training, thereby mitigating the cumulative drift errors inherently introduced by inertial integration.

### 2.3. UAV Kinematic Model

Based on the IMU measurement model, the continuous-time kinematic equations describing the evolution of the UAV state are given as follows [18,33]. The state consists of position $p^{W}\left(t\right)$, velocity $v^{W}\left(t\right)$, and attitude quaternion $q_{WB}\left(t\right)$. For the translational component, the following holds:

(11) $${\dot{p}}^{W}\left(t\right)=v^{W}\left(t\right),$$

(12) $${\dot{v}}^{W}\left(t\right)=a^{W}\left(t\right),$$

The rotational motion is driven by angular velocity, and the quaternion differential equation can be written as follows:

(13) $${\dot{q}}_{WB}\left(t\right)=\frac{1}{2}\Omega \left(\omega^{B}\left(t\right)\right)q_{WB}\left(t\right),$$

where ω(t) is the quaternion multiplication matrix constructed from angular velocity:

(14) $$\Omega \left(\omega \right)=\left[\begin{array}{cccc} 0 & -\omega_{x} & -\omega_{y} & -\omega_{z}\\ \omega_{x} & 0 & \omega_{z} & -\omega_{y}\\ \omega_{y} & -\omega_{z} & 0 & \omega_{x}\\ \omega_{z} & \omega_{y} & -\omega_{x} & 0 \end{array}\right],$$

considering discrete IMU sampling with sampling interval Δt, the discrete-time index is denoted by k. From the estimated attitude, the rotation matrix $R_{WB}(q_{k})$ is obtained. By transforming the body-frame specific force into the world frame and adding gravity, the world-frame acceleration is written as follows:

(15) $$a_{k}^{W}\left(t\right)=R_{WB}\left(q_{k}\right)\left(f_{m,k}^{B}-b_{a,k}\right)+g^{W},$$

Applying first-order integration yields the velocity recursion:

(16) $$v_{k+1}^{W}=v_{k}^{W}+a_{k}^{W}\Delta t,$$

And the position update equation becomes the following:

(17) $$p_{k+1}^{W}=p_{k}^{W}+v_{k}^{W}\Delta t+\frac{1}{2}a_{k}^{W}\Delta t,$$

In discrete time, the quaternion is updated using a first-order approximation under the small-angle assumption, followed by normalisation to preserve its unit norm. Let

(18) $$\omega_{k}^{B}=\omega_{m,k}^{B}-b_{g,k},$$

Then we obtain the following:

(19) $$\delta q_{k}\approx \left[\begin{array}{c} 1\\ \frac{1}{2}\omega_{K}^{B}\Delta t \end{array}\right],q_{k+1}=norm\left(q_{k}⊗\delta q_{k}\right),$$

where $norm\left(\cdot \right)$ denotes the quaternion normalisation operation.

The small-angle assumption applies exclusively to the local rotational increments between consecutive IMU samples and therefore imposes no restriction on the large-angle global attitude or dynamic manoeuvres of the UAV. Based on the above discrete-time inertial kinematic relationships, the IMU-driven state-propagation dependency can be described as follows: the gyroscope angular velocity drives attitude propagation; the updated attitude determines the rotation matrix; the rotation matrix transforms the body-frame specific force into the world frame to obtain the world-frame acceleration; and finally, this acceleration is integrated to update the velocity and position. The mathematical formulations corresponding to this dependency have already been given in Equations (15)–(19).

Leveraging these classical discrete-time inertial kinematic relationships, the devised loss functions incorporate consistency for attitude propagation, acceleration coordinate transformation, and velocity–position integration. These well-established physical principles are integrated into the end-to-end learning framework as differentiable regularisation.

## 3. Time-Series Feature Modelling Based on Long Short-Term Memory Networks

### 3.1. Long Short-Term Memory Networks

Long short-term memory (LSTM) networks are a class of recurrent neural network (RNN) architectures for sequence modelling [34,35]. By introducing gating mechanisms into the recurrent structure, LSTMs enable the selective retention and updating of information, allowing them to capture both short-term dynamics and long-term dependencies while alleviating the vanishing-gradient problem encountered by traditional RNNs during long-sequence training.

**Figure 2:**
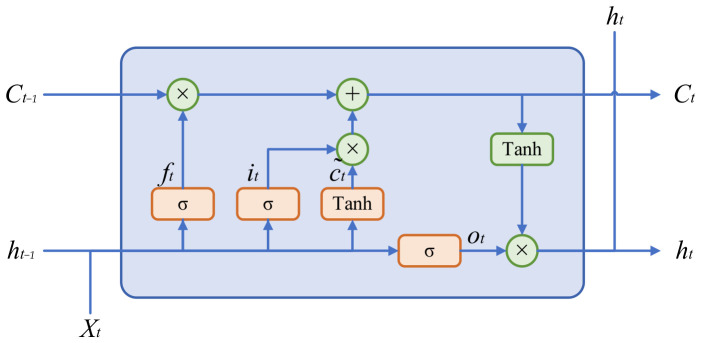
Schematic diagram of the LSTM structure.

An LSTM consists of a forget gate, an input gate, and an output gate, which jointly participate in the update of the memory cell and enable the network to selectively preserve and update historical information over time. Let the input at time t be $x_{t}$, the hidden state be $h_{t}$, and the cell state be $c_{t}$. Here, $\sigma \left(\cdot \right)$ denotes the sigmoid activation function, $\tanh\left(\cdot \right)$ denotes the hyperbolic tangent function. The standard LSTM update equations are given as follows:

(20) $$f_{t}=\sigma \left(W_{f}\left[h_{t-1},x_{t}\right]+b_{f}\right),$$

(21) $$i_{t}=\sigma \left(W_{i}\left[h_{t-1},x_{t}\right]+b_{i}\right),$$

(22) $${\tilde{c}}_{t}=\tanh\left(W_{c}\left[h_{t-1},x_{t}\right]+b_{c}\right),$$

(23) $$c_{t}=f_{t}*c_{t-1}+i_{t}*{\tilde{c}}_{t},$$

(24) $$o_{t}=\sigma \left(W_{o}\left[h_{t-1},x_{t}\right]+b_{o}\right),$$

(25) $$h_{t}=o_{t}*\tanh\left(c_{t}\right),$$

where the forget gate $f_{t}$ controls the extent to which the historical memory $c_{t-1}$ is retained, the input gate $i_{t}$ controls the amount of new information written at the current time step, $c_{t}$ denotes the candidate memory generated from the current input and the previous hidden state, and the output gate $o_{t}$ determines which memory components are exposed as the hidden state. This gating mechanism enables the network to continuously retain and retrieve key dynamic information even in the presence of noise and disturbances. The basic structure of an LSTM is shown in Figure 2.

### 3.2. Modelling Temporal Features Using Long Short-Term Memory Networks

To establish temporal dependencies within IMU sequences, this paper employs an LSTM-based temporal encoder to model the dynamic information contained within each input time window. Through its gating mechanism, the encoder captures both short-term dynamics and long-term dependencies, thereby improving the representation of complex manoeuvring patterns [35].

For a time window of length T, the input sequence is defined as follows:

(26) $$U_{k:k+T-1}=\left\{u_{k},u_{k+1},\cdots ,u_{k+T-1}\right\},$$

where $u_{k}$ denotes the IMU observation at time step k. In this modelling framework, the hidden state at the end of the window, $h_{T}$, is treated as a compact representation of the temporal features within the current time window [24]. This terminal hidden state provides the shared feature basis for subsequent structured regression of the position-and-attitude state. If a direct state mapping is considered, the temporal feature can be projected into the position-and-attitude state space through a fully connected transformation:

(27) $$s_{T}=W_{h}h_{T}+b,$$

where $s_{T}$ denotes the position-and-attitude state at the end of the window. In the present study, this temporal representation is further used as the shared encoded feature for the subsequent dual-branch regression framework.

To ensure that the attitude output represents a valid rotation, the predicted quaternion is normalised as follows:

(28) $${\hat{q}}_{WB,T}\left(t\right)=\frac{{\hat{q}}_{WB,T}}{\left\|{\hat{q}}_{WB,T}\right\|},$$

After this normalisation, the predicted quaternion satisfies the unit-norm constraint and therefore corresponds to a valid three-dimensional rotation. This normalisation helps avoid invalid attitude outputs caused by numerical deviations. The above temporal feature extraction framework provides a unified representation of flight manoeuvres and serves as the basis for the subsequent dual-branch structured regression model.

## 4. Physical Consistency Constraints Based on Physics-Informed Learning

### 4.1. Physics-Informed Neural Networks

**Figure 3:**
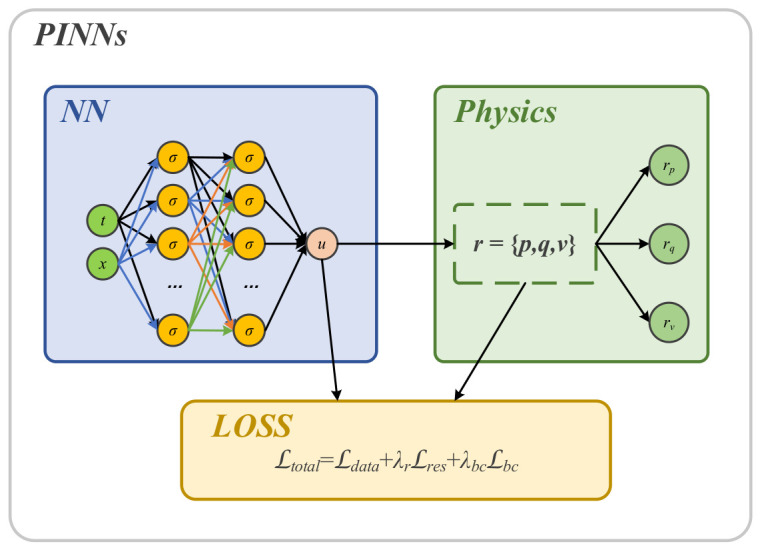
Schematic diagram of the PINN framework.

Physics-informed neural networks (PINNs) [36] are a class of learning frameworks that explicitly incorporate known physical laws into the training process. Their core idea is to embed physical prior knowledge—such as governing equations, conservation laws, and boundary or initial conditions—into the loss function or network structure in the form of constraints, so that the network output satisfies both data consistency and physical consistency during training and inference [37]. Generally, the training objective of PINNs can be written as a weighted combination of a data-fitting term, a physical residual term, and a boundary-condition term:

(29) $$ℒ={ℒ}_{data}+\lambda_{r}{ℒ}_{res}+\lambda_{bc}{ℒ}_{bc},$$

where ${ℒ}_{data}$ measures the discrepancy between the network output and the observed data, ${ℒ}_{res}$ denotes the residual loss obtained by substituting the network prediction into the physical governing equations, and ${ℒ}_{bc}$ denotes the boundary-condition loss. PINNs convert physical equations or kinematic constraints into trainable supervisory signals in the optimisation objective, thereby restricting the learned solution to the physically feasible domain and improving interpretability. In addition, physical consistency constraints can suppress non-physical fluctuations and the accumulation of long-term errors, thereby reducing drift in long-term prediction tasks. As physical laws are, to some extent, independent of specific data distributions [38], physics-informed learning methods often provide more stable inductive biases and may improve stability under data-distribution shifts. A schematic diagram of a general PINN framework is shown in Figure 3.

### 4.2. Physics-Informed Constraint Modelling

To improve model stability under complex manoeuvres and external disturbances, this study draws on the core idea of physics-informed learning and introduces physics-informed consistency constraints during training [28]. By embedding established inertial kinematic relationships into the optimisation objective, the network is encouraged to satisfy fundamental motion constraints while fitting the labelled data.

To this end, several types of physical consistency constraints are incorporated into the loss function, including quaternion consistency, acceleration coordinate transformation consistency, velocity integration consistency, position integration consistency, and vector transformation consistency. These constraints ensure that the model outputs remain physically plausible and reduce the risk of overfitting to the label distribution of specific scenarios. The specific constraint terms are defined as follows:

Quaternion attitude consistency:

(30) $$L_{quat}^{\left(k\right)}={\left\|q_{WB,k+1}-\frac{q_{WB,k}⊗\delta q_{k}}{\left\|q_{WB,k}⊗\delta q_{k}\right\|}\right\|}_{2}^{2},$$

Acceleration coordinate transformation consistency:

(31) $$L_{acc}^{\left(k\right)}={\left\|R_{WB}\left(q_{WB,k}\right)\cdot \left(f_{m,k}^{B}-b_{a,k}\right)+g^{W}-a_{k}^{W}\right\|}_{2}^{2},$$

Velocity integration consistency:

(32) $$L_{vel}^{\left(k\right)}={\left\|v_{k+1}^{W}-\left(v_{k}^{W}+a_{k}^{W}\Delta t\right)\right\|}_{2}^{2},$$

Position integration consistency:

(33) $$L_{pos}^{\left(k\right)}={\left\|p_{k+1}^{W}-\left(p_{k}^{W}+v_{k}^{W}\Delta t+\frac{1}{2}a_{k}^{W}\Delta t^{2}\right)\right\|}_{2}^{2},$$

Vector transformation consistency:

(34) $$L_{vec}^{\left(k\right)}={\left\|R_{WB}\left(q_{WB,k}\right)\cdot v_{k}^{B}-v_{k}^{W}\right\|}_{2}^{2},$$

The above residual terms are selected according to the discrete inertial kinematic chain used in this study. The quaternion consistency term corresponds to angular-velocity-driven attitude propagation, the acceleration transformation term links body-frame specific force to world-frame acceleration through the estimated attitude, and the velocity and position integration terms regularise the translational recursion. The vector transformation term further constrains the geometric consistency between attitude and kinematic quantities. These constraints are derived from the state-propagation relationships formulated in Section 2. The main functions of the above physical consistency constraints are as follows:

1. The quaternion consistency constraint is used to regularise the angular-velocity-driven attitude recursion and suppress non-physical jumps.
2. The kinematic integration consistency constraints, including velocity and position integration, are used to enforce the recursive relationships among acceleration, velocity, and position, thereby reducing cumulative error.
3. The transformation consistency constraint ensures consistency between the body-frame specific force and the world-frame acceleration through attitude-based rotation and gravity compensation.
4. The vector transformation consistency constraint is used to regularise the geometric relationship between attitude and kinematic quantities.

Accordingly, the overall training objective is formulated as the weighted sum of the data supervision term and the physical consistency:

(35) $$L_{total}=L_{data}+\lambda_{acc}L_{acc}+\lambda_{vel}L_{vel}+\lambda_{pos}L_{pos}+\lambda_{quat}L_{quat}+\lambda_{vec}L_{vec},$$

where $\lambda_{acc}$, $\lambda_{vel}$, $\lambda_{pos}$, $\lambda_{quat}$ and $\lambda_{vec}$ are the weighting coefficients of the corresponding physical terms. By embedding these physical consistency constraints into the learning process [37], the proposed framework explicitly incorporates kinematic priors into the end-to-end training objective. Consequently, the network is guided toward physically consistent predictions, effectively mitigating integration-induced drift and enhancing estimation stability.

The weighting coefficients of the physical consistency are fixed during training and selected based on validation-set performance and numerical stability. In this study, the data-fitting loss is kept as the dominant supervision term, while the physical consistency losses are used as auxiliary regularisation. This setting is adopted because different physical residuals have different units, numerical scales, and sensitivities to IMU noise, bias, and integration errors. Acceleration- and integration-related residuals can be strongly affected by inertial measurement noise and numerical differentiation. Relatively small weights are assigned to these terms to prevent them from dominating the optimisation process, while still encouraging the predicted motion states to satisfy the established inertial kinematic relationships.

The proposed physical consistency constraints are derived from classical inertial kinematic relationships, serving as a regularisation mechanism during supervised optimization. In practice, deviations from these mathematical relationships inevitably arise from both network prediction errors and inherent IMU imperfections, such as measurement noise, bias, scale-factor errors, or sensor saturation. Crucially, these constraints do not directly eliminate raw sensor errors; instead, they regularise the predicted motion states by enforcing mathematical coherence across the attitude propagation, acceleration transformation, and velocity–position integration chain. This formulation imparts a layer of physical interpretability to the optimization path of the otherwise black-box deep learning model. By grounding position and attitude predictions within explicit kinematic laws, the estimated states adhere to underlying mechanical principles rather than relying solely on an unconstrained data-driven mapping from IMU inputs. Consequently, the network learns to suppress cumulative tracking errors induced by noisy and biased inertial data while preserving the physical coherence of the estimated trajectories.

## 5. DPI-LSTM: A Dual-Branch Physics-Informed LSTM Model for UAV Position and Attitude Estimation

### 5.1. Model Architecture

To unify temporal feature modelling and physical consistency constraints in position and attitude estimation, this paper proposes a dual-branch physics-informed architecture based on shared temporal encoding and physical-link coupling. The architecture first extracts shared temporal features of flight dynamics from IMU sequences through a common temporal encoder and then models translational and rotational states separately through the position branch and the attitude branch. By incorporating physical consistency constraints during training, the model enforces mathematical coherence between translational and rotational state estimates based on classical inertial kinematics, thereby establishing a unified end-to-end learning framework comprising shared temporal encoding, dual-branch structured regression, and explicit kinematic coupling.

Specifically, the network takes IMU time-window sequences as input and uses an LSTM encoder to extract temporal feature representations:

(36) $$h_{T}=LSTM\left(U_{k:k+T-1}\right),$$

The hidden state at the end of the window, $h_{T}$, is regarded as a compact representation of the UAV’s temporal dynamics within the current time window and is used to characterise manoeuvring patterns and sensor variation characteristics over that interval.

Accordingly, the network leverages a dual-branch architecture to regress translational and rotational states via dedicated position and attitude branches, respectively. This design draws inspiration from multi-task state-estimation networks [29,30], where rotation and translation are treated as coupled yet distinct prediction targets branching from a shared feature representation. The position branch takes the shared temporal feature $h_{T}$ as input and maps it through a fully connected layer to obtain the position prediction in the world frame, as follows:

(37) $${\hat{p}}_{T}^{W}=f_{pos}\left(h_{T}\right),$$

The attitude branch is responsible for regressing the quaternion at the end of the window. To enforce the unit–quaternion constraint, the predicted quaternion is normalised to obtain the final attitude estimate:

(38) $${\hat{q}}_{WB,T}=\frac{{\hat{q}}_{WB,T}}{\left\|{\hat{q}}_{WB,T}\right\|},$$

Once the attitude estimate has been obtained, the corresponding rotation matrix $R_{WB}\left({\hat{q}}_{WB,T}\right)$ is used to transform the body-frame specific force into the world frame, thereby yielding the acceleration required for the subsequent physical consistency constraints:

(39) $${\hat{a}}_{T}^{W}=R_{WB}\left({\hat{q}}_{WB,T}\right)\left(f^{B}\left(t\right)-b_{a,T}\right)+g^{W},$$

The predicted acceleration is further involved in the velocity and position integration constraints:

(40) $${\hat{v}}_{T+1}^{W}={\hat{v}}_{T}^{W}+{\hat{a}}_{T}^{W}\Delta t,$$

(41) $${\hat{p}}_{T+1}^{W}={\hat{p}}_{T}^{W}+{\hat{v}}_{T}^{W}\Delta t+\frac{1}{2}{\hat{a}}_{T}^{W}\Delta t^{2},$$

During training, these established inertial kinematic relationships are used to construct consistency losses linking the attitude output, acceleration transformation, and velocity–position integration [37]. The attitude branch is primarily responsible for learning rotational dynamics, while also influencing the consistency of translational state learning through coordinate transformations and integration relationships. Concurrently, the position branch models translational dynamics based on the shared temporal features, while its cross-branch coherence with the attitude component is regularised through physical consistency constraints, thereby integrating the learning of rotational and translational states into a structured architecture and further enhancing the long-term stability of position estimation.

Compared with a single-branch network with fully shared parameters, the proposed dual-branch physical-link coupling structure has the following advantages:

1. Reduced multi-task gradient interference.

By regressing attitude and position through separate task-specific branches, the architecture effectively mitigates cross-task interference between distinct regression objectives, while the shared LSTM encoder captures underlying temporal motion characteristics. This design closely aligns with the fundamental paradigm of multi-task learning, which utilises shared feature representations coupled with task-specific prediction heads.

2. Improved modelling of dynamical consistency.

By strengthening physical consistency constraints derived from classical inertial kinematics, the model promotes mathematical coherence between rotational and translational state estimates, thereby enhancing the stability of attitude and position tracking under highly manoeuvrable flight conditions.

**Figure 4:**
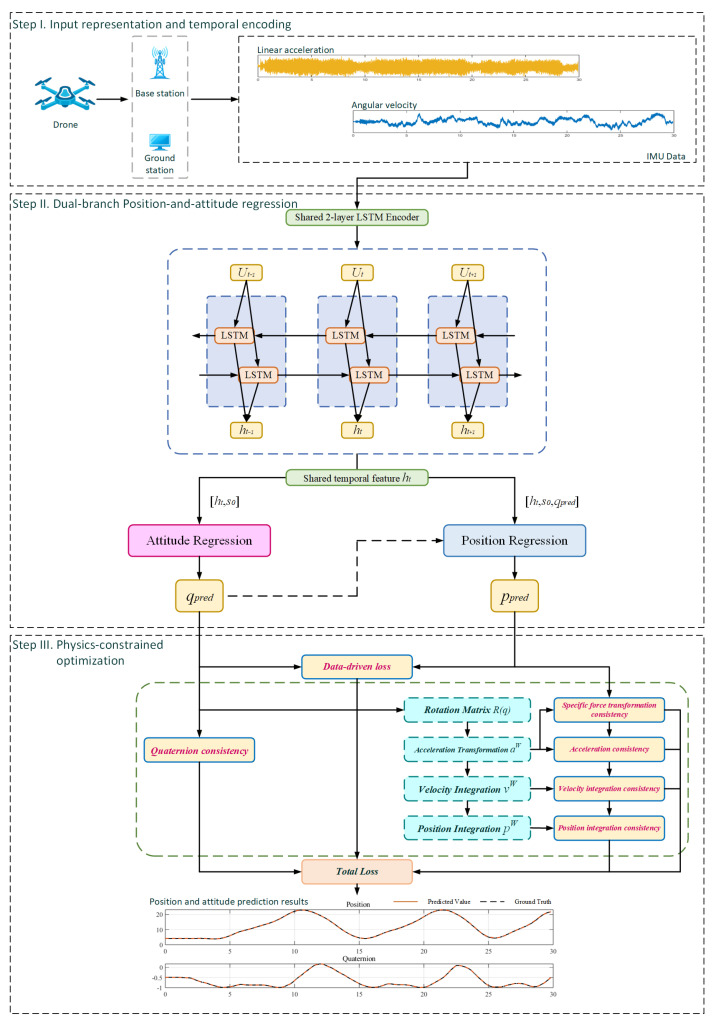
Overall architecture of the proposed DPI-LSTM model.

Consequently, this paper establishes a unified modelling framework that integrates shared temporal encoding, dual-branch state regression, and physical consistency constraints, with the overall structure shown in Figure 4.

### 5.2. Evaluation Metrics

To comprehensively characterise the performance of the proposed model in UAV position and attitude estimation, this paper adopts the mean squared error (*MSE*), root mean square error (*RMSE*), mean absolute error (*MAE*), and coefficient of determination (*R*^2^) as evaluation metrics. Among them, *MSE* is more sensitive to large deviations and can reflect abnormal errors such as abrupt trajectory changes or drift. *RMSE* has the same physical dimension as the original quantity and therefore provides good engineering interpretability. *MAE* is relatively more robust to outliers and can more stably reflect the average deviation level of the predictions. The *R*^2^ is used to measure the degree to which the model fits the variation trend of the ground-truth data.

Since the UAV state includes both position and attitude, model performance is evaluated from three perspectives: position error, attitude error, and error distribution. The position metrics are used to measure the accuracy of estimating the UAV trajectory in the world frame and reflect the model’s capability in translational motion estimation. The attitude metrics are used to assess the prediction accuracy of rotational motion. During training, attitude is represented using quaternions to avoid the singularity problem associated with Euler angles and to improve numerical stability; during evaluation, the predicted quaternions are converted into Euler angles to obtain attitude error metrics with more explicit physical meaning. In addition, a combined error distribution analysis is introduced to provide a statistical description of the overall distribution characteristics of both position and attitude errors, thereby offering a more comprehensive reflection of the model’s practical performance in digital twin state mapping tasks.

Assume that there are N time samples. The predicted and ground-truth position and attitude are denoted by ${\hat{p}}^{W}\left(t_{i}\right)$, $p^{W}\left(t_{i}\right)$, ${\hat{q}}_{WB}\left(t_{i}\right)$ and $q_{WB}\left(t_{i}\right)$, respectively. The quaternion outputs are first converted into Euler angles. The roll, pitch, and yaw angles are computed as follows:

(42) $$\phi =a\tan2\left(2\left(q_{w}q_{x}+q_{y}q_{z}\right),1-2\left({q_{x}}^{2}+{q_{y}}^{2}\right)\right),$$

(43) $$\theta =\arcsin\left(2\left(q_{w}q_{y}-q_{z}q_{x}\right)\right),$$

(44) $$\psi =a\tan2\left(2\left(q_{w}q_{z}+q_{x}q_{y}\right),1-2\left({q_{y}}^{2}+{q_{z}}^{2}\right)\right),$$

Accordingly, the Euler-angle attitude vector is defined as follows:

(45) $$e\left(t_{i}\right)={\left[\phi \left(t_{i}\right),\theta \left(t_{i}\right),\psi \left(t_{i}\right)\right]}^{T},$$

The position evaluation metrics are defined as follows:

(46) $$MSE_{pos}=\frac{1}{N}{\sum_{i=1}^{N}\left\|{\hat{p}}^{W}\left(t_{i}\right)-p^{W}\left(t_{i}\right)\right\|}_{2}^{2},$$

(47) $$RMSE_{pos}=\sqrt{MSE_{pos}},$$

(48) $$MAE_{pos}=\frac{1}{N}{\sum_{i=1}^{N}\left\|{\hat{p}}^{W}\left(t_{i}\right)-p^{W}\left(t_{i}\right)\right\|}_{2},$$

(49) $${R^{2}}_{pos}=1-\frac{\sum_{i=1}^{N}{\left\|{\hat{p}}^{W}\left(t_{i}\right)-p^{W}\left(t_{i}\right)\right\|}_{2}^{2}}{\sum_{i=1}^{N}{\left\|p^{W}\left(t_{i}\right)-{\bar{p}}^{W}\left(t_{i}\right)\right\|}_{2}^{2}},\;{\bar{p}}^{W}\left(t_{i}\right)=\frac{1}{N}\sum_{i=1}^{N}p^{W}\left(t_{i}\right),$$

Attitude evaluation metrics include the following:

(50) $$MSE_{att}=\frac{1}{N}{\sum_{i=1}^{N}\left\|\hat{e}\left(t_{i}\right)-e\left(t_{i}\right)\right\|}_{2}^{2},$$

(51) $$RMSE_{att}=\sqrt{MSE_{att}},$$

(52) $$MAE_{att}=\frac{1}{N}{\sum_{i=1}^{N}\left\|\hat{e}\left(t_{i}\right)-e\left(t_{i}\right)\right\|}_{2},$$

(53) $${R^{2}}_{att}=1-\frac{\sum_{i=1}^{N}{\left\|\hat{e}\left(t_{i}\right)-e\left(t_{i}\right)\right\|}_{2}^{2}}{\sum_{i=1}^{N}{\left\|e\left(t_{i}\right)-\bar{e}\left(t_{i}\right)\right\|}_{2}^{2}},\;{\bar{e}}^{W}\left(t_{i}\right)=\frac{1}{N}\sum_{i=1}^{N}p^{W}\left(t_{i}\right),$$

Furthermore, to better reflect the overall error characteristics of the model in joint position and attitude estimation, the position error, the attitude error, and the combined error derived from both are defined as follows:

(54) $$e_{pos}\left(t_{i}\right)={\left\|{\hat{p}}^{W}\left(t_{i}\right)-p^{W}\left(t_{i}\right)\right\|}_{2},$$

(55) $$e_{att}\left(t_{i}\right)=2\arccos\left(\left|{\hat{q}}_{WB}\left(t_{i}\right)-q_{WB}\left(t_{i}\right)\right|\right)\times \frac{180}{\pi },$$

(56) $$e_{comp}\left(t_{i}\right)=\frac{e_{pos}\left(t_{i}\right)}{c_{pos}}+\frac{e_{att}\left(t_{i}\right)}{c_{att}},$$

where $c_{pos}$ and $c_{att}$ are the normalisation constants for position error and attitude error, respectively. The cumulative distribution functions (CDFs) of these three errors are analysed to characterise the proportion of samples falling within different error thresholds. A higher cumulative probability at a smaller error threshold indicates that the model has a more concentrated error distribution and better estimation stability, thereby reflecting its robustness in joint position and attitude estimation.

## 6. Experimental Results and Analysis

### 6.1. Dataset and Experimental Setup

This study uses the UZH-FPV Drone Racing dataset [39] released by the Robotics and Perception Group of the University of Zurich (UZH). The dataset contains more than 27 flight sequences, covering a total flight distance of over 10 km and including both indoor and outdoor racing environments. It provides synchronised IMU and camera data, as well as high-precision six degrees of freedom (6-DoF) ground-truth annotations, including position and attitude; however, only the IMU data and the corresponding position and attitude annotations are used in this study. The flight sequences are characterised by high manoeuvrability and rapid variations in acceleration and angular velocity, which pose significant challenges for state estimation and model learning. Ground-truth position and attitude annotations were obtained from external measurements using an MS60 laser tracker (Leica Geosystems AG, Heerbrugg, Switzerland).

For a fair comparison, all learning-based methods were evaluated under the same sequence-level data split and the same evaluation metrics. The models were trained and tested using the same IMU inputs and position and attitude labels, and no test sequence was used during training or validation. The hyperparameters of the compared learning-based methods were selected based on validation-set performance under the same experimental protocol. The traditional inertial-navigation baselines were evaluated using the same IMU measurements and initial reference state.

In this study, the Snapdragon Flight IMU stream from the UZH-FPV Drone Racing dataset is used. The IMU data were recorded from the Snapdragon Flight board (Qualcomm Technologies, Inc., San Diego, CA, USA). The IMU measurements are sampled at 500 Hz, corresponding to a sampling interval of 0.002 s, and only the three-axis gyroscope and three-axis accelerometer data are used as model inputs. The dataset-provided reference trajectory is generated using external Leica MS60 position measurements together with batch optimisation. The main specifications of the Snapdragon Flight IMU used in this study are summarised in Table 1.

To clarify the main architectural settings and physical-loss configuration of the proposed DPI-LSTM, the key implementation details are summarised in Table 2. The physical consistency losses are used as auxiliary regularisation losses, and their weights are fixed during training to encourage kinematic consistency while avoiding domination of the optimisation process by scale-sensitive residuals.

In the experiments, IMU data are used as input and ground-truth position and attitude as supervision to train an end-to-end IMU-to-position-and-attitude regression model. During inference, the trained model estimates the UAV position and attitude directly from IMU time-window sequences. To avoid data leakage, all samples are split on a sequence basis into training, validation, testing, and cross-sequence evaluation sets. Specifically, four indoor sequences are used for training, while two additional indoor sequences are used for validation and testing, respectively. One outdoor sequence is further reserved for cross-sequence evaluation. After sequence-wise splitting and preprocessing, the numbers of samples in the training, validation, and test sets are approximately in the ratio of 8:1:1. The outdoor sequence is used for cross-sequence evaluation within the UZH-FPV dataset, while all sets remain strictly non-overlapping in both time and sequence. For illustration, Table 3 presents sample data from one flight sequence in the UZH-FPV dataset.

**Figure 5:**
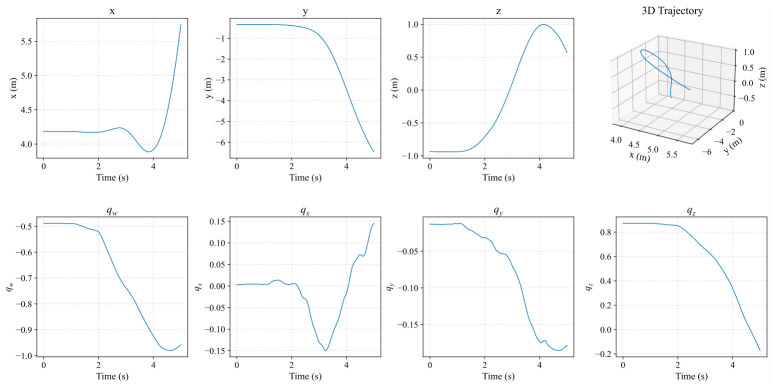
Time–series curves of position and quaternion components in an example sequence segment.

Each row corresponds to one observation sample at a specific time and contains two groups of information: the reference position and attitude labels, namely the position $p^{W}\left(t\right)$ and attitude quaternion $q_{WB}\left(t\right)$ in the world frame, and the IMU inputs, namely the angular velocity vector $\omega^{B}\left(t\right)$ and the specific force vector $f^{B}\left(t\right)$ in the body frame. Furthermore, the timestamps have been converted to relative time for convenience in processing. This conversion does not affect the sampling interval or the underlying motion dynamics. Figure 5 provides an illustrative example of the position and attitude variations within a flight sequence segment.

### 6.2. Comparative Methods

To comprehensively evaluate the effectiveness of the proposed method, this study compares it with traditional inertial navigation methods and representative deep learning methods under identical data splits and evaluation metrics. The traditional methods include a direct inertial navigation system (Direct INS), a bias-calibrated inertial navigation system (Bias INS) [18], and a Mahony-filter-based inertial navigation system (Mahony INS) [40]. These methods mainly rely on integration or filtering of IMU measurements for state estimation. Although they have clear physical structures, they are highly susceptible to noise and sensor bias in the absence of external observations, leading to cumulative errors and significant drift over time.

The deep learning methods include IONet [24], CNN–LSTM [26], and RoNIN [25]. These methods learn the mapping from IMU sequences to motion states in a data-driven manner and can mitigate inertial drift to some extent. However, because they lack explicit physical constraints, their outputs may still exhibit insufficient physical consistency in long-duration sequences and highly dynamic scenarios. DPI-LSTM incorporates established inertial kinematic relationships as physical consistency loss terms within an end-to-end learning framework, thereby regularising the consistency between translational and rotational state estimates.

**Figure 6:**
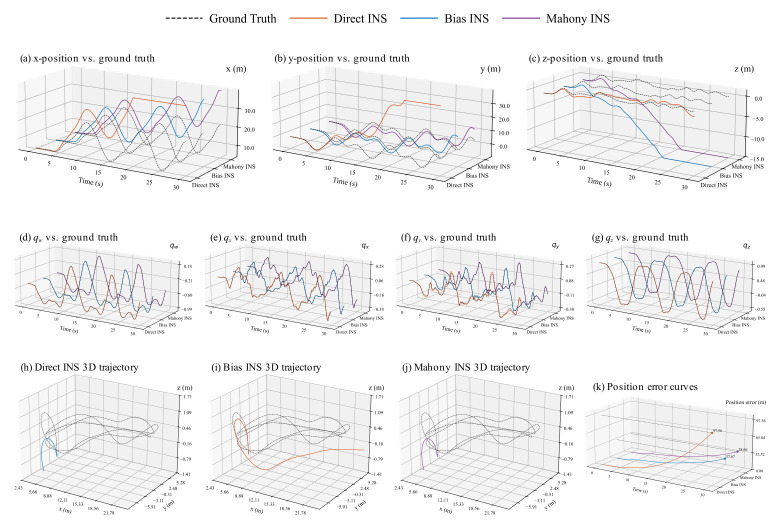
Qualitative comparison of position and attitude reconstruction and error evolution for traditional inertial navigation methods: (**a**–**c**) position estimates along the x-, y-, and z-axes versus the ground truth; (**d**–**g**) quaternion estimates *q_w_*, *q_x_*, *q_y_*, and *q_z_* versus the ground truth; (**h**–**j**) 3D trajectory comparisons of Direct INS, Bias INS, and Mahony INS with the ground truth; (**k**) position error curves of different methods.

**Figure 7:**
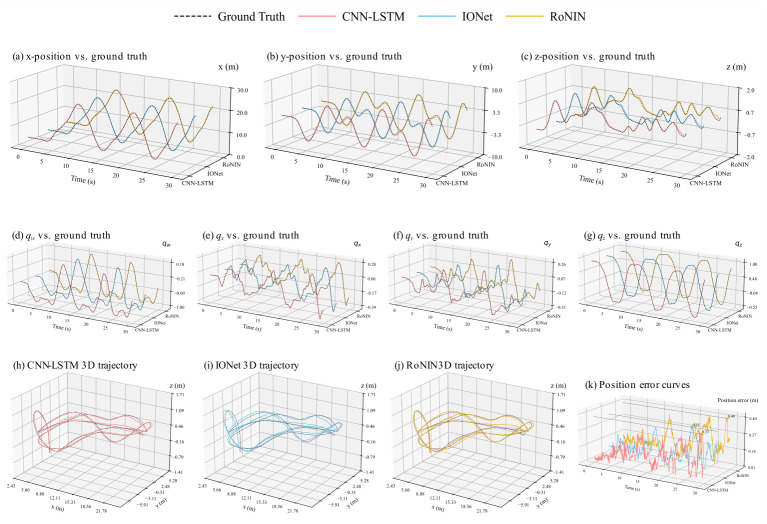
Qualitative comparison of position and attitude reconstruction and error evolution for deep learning-based methods: (**a**–**c**) position estimates along the x-, y-, and z-axes versus the ground truth; (**d**–**g**) quaternion estimates *q_w_*, *q_x_*, *q_y_*, and *q_z_* versus the ground truth; (**h**–**j**) 3D trajectory comparisons of CNN-LSTM, IONet, and RoNIN with the ground truth; (**k**) position error curves of different methods.

**Figure 8:**
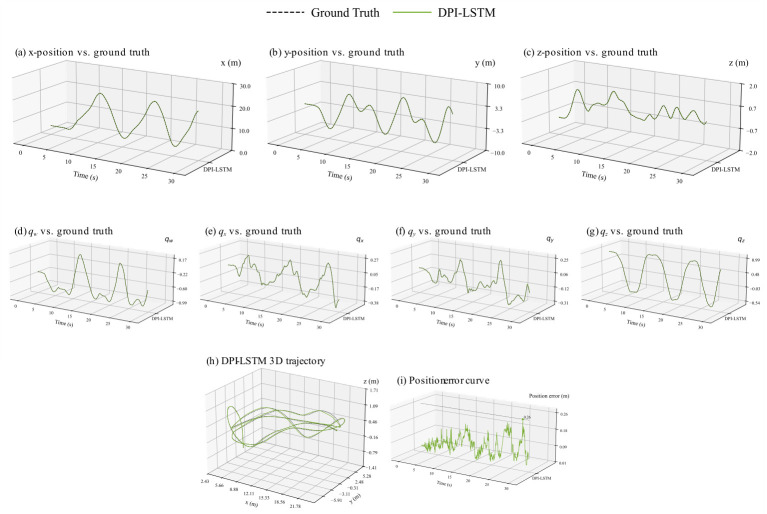
Qualitative comparison of position and attitude reconstruction and error evolution for the proposed DPI-LSTM method: (**a**–**c**) position estimates along the x-, y-, and z-axes versus the ground truth; (**d**–**g**) quaternion estimates *q_w_*, *q_x_*, *q_y_*, and *q_z_* versus the ground truth; (**h**) 3D trajectory comparison of DPI-LSTM with the ground truth; (**i**) position error curve of the proposed method.

Figure 6, Figure 7 and Figure 8 present a qualitative comparison of the estimated position and attitude states, as well as the error evolution, of different methods. The results reveal clear differences among the compared methods in terms of trajectory reconstruction and error evolution. For the traditional inertial navigation methods, namely Direct INS, Bias INS, and Mahony INS, significant discrepancies can be observed in the position–component curves, as shown in Figure 6a–c, and in the three-dimensional (3D) trajectory plots, as shown in Figure 6h–j. As time progresses, the predicted trajectories gradually deviate from the ground-truth trajectories, indicating the characteristic drift of pure inertial integration.

Deep learning methods generally reconstruct the ground-truth trajectories more accurately, indicating that data-driven models can learn more effective motion representations from historical IMU sequences. IONet, CNN–LSTM, and RoNIN clearly outperform the traditional methods in both the position–component curves, as shown in Figure 7a–c, and the 3D trajectories, as shown in Figure 7h–j. However, noticeable lag or local deviations still occur in segments involving rapid state changes or frequent motion transitions.

In comparison, the predicted trajectory of DPI-LSTM shows the highest degree of alignment with the ground-truth trajectory, and its position component curves more closely follow the ground-truth values in both global trends and local details. This indicates that the introduced physical consistency constraints and dual-branch coupling mechanism can more effectively suppress cumulative drift, thereby improving the stability and accuracy of position estimation. In the quaternion comparison plots, namely Figure 6d–g, Figure 7d–g and Figure 8d–g, the attitude outputs of the compared methods are generally close to the ground truth, while DPI-LSTM maintains better smoothness and consistency in attitude estimation. In addition, the position–error curve of DPI-LSTM, shown in Figure 8i, remains at a relatively low level compared with the error curves of the other methods shown in Figure 6k and Figure 7k.

**Figure 9:**
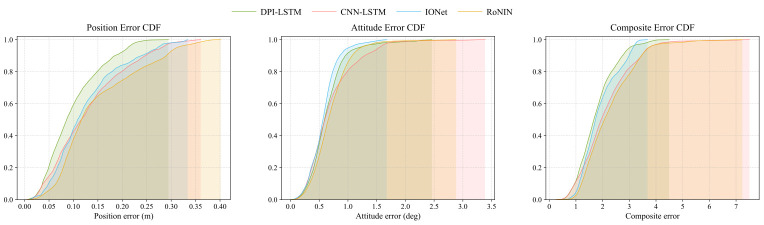
CDF comparison of position, attitude, and combined errors among deep learning methods and DPI-LSTM.

Figure 9 shows that the error distribution of DPI-LSTM is generally more concentrated, with its CDF reaching higher cumulative probabilities at smaller error thresholds across most intervals. This indicates that a larger proportion of its estimation errors are controlled within a smaller range, further demonstrating the superiority of the proposed method in terms of stability and robustness.

Table 4 summarises the quantitative test results of all compared methods. The traditional methods perform substantially worse in terms of position accuracy. In particular, the position *RMSE* values of Bias INS and Mahony INS reach 9.60 m and 9.91 m, respectively, indicating that, in the absence of external observations, traditional methods struggle to suppress the accumulation of position errors. In contrast, deep learning methods substantially reduce position error, with IONet, CNN–LSTM, and RoNIN achieving position *RMSE* values of 0.0831 m, 0.0869 m, and 0.0980 m, respectively, demonstrating the effectiveness of data-driven modelling for inertial position and attitude estimation.

Building on this, DPI-LSTM achieves the best performance in position estimation, with *MSE_pos_* = 0.00428 m^2^, *RMSE_pos_* = 0.0654 m, and *MAE_pos_* = 0.0484 m, while *R*^2^*_pos_* reaches 0.998. These results outperform all competing methods on the position metrics, indicating that the proposed method provides the strongest performance in position estimation. Regarding the attitude metrics, DPI-LSTM achieves *MSE_att_* = 5.62 × 10^−5^, *RMSE_att_* = 0.00749, and *MAE_att_* = 0.00577. While attitude estimation remains highly accurate, the main performance gains are concentrated in position accuracy and in the suppression of long-term drift.

Overall, DPI-LSTM improves position-estimation accuracy while maintaining stable and accurate attitude estimation, thereby validating the effectiveness of the proposed physical consistency constraints and dual-branch architecture under complex motion conditions.

### 6.3. Ablation Experiments

To further analyse the contribution of each key design element to performance improvement, ablation experiments are conducted under a unified experimental setup. By progressively introducing or removing the dual-branch structure and the physical consistency constraints, the study systematically evaluates the effect of different modules on position and attitude estimation performance.

The ablation variants include the baseline LSTM, physics-informed long short-term memory (PI-LSTM), dual-branch long short-term memory (D-LSTM), and the complete DPI-LSTM.

**Figure 10:**
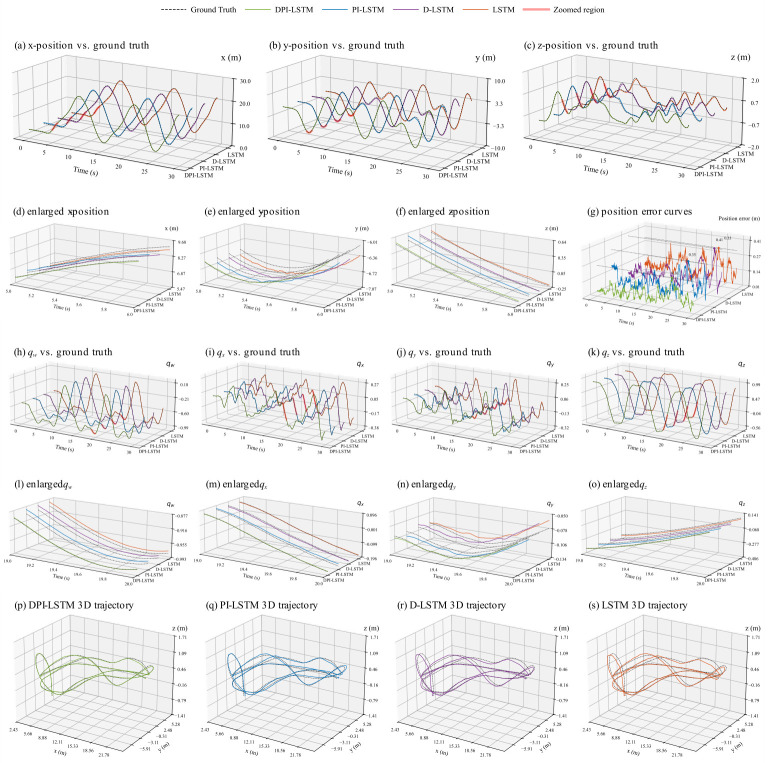
Qualitative comparison of position and attitude estimation results and errors in the ablation experiments: (**a**–**c**) position estimates along the x-, y-, and z-axes versus the ground truth; (**d**–**f**) enlarged views of the x-, y-, and z-position estimates; (**g**) position error curves of different methods; (**h**–**k**) quaternion estimates *q_w_*, *q_x_*, *q_y_*, and *q_z_* versus the ground truth; (**l**–**o**) enlarged views of the quaternion estimates *q_w_*, *q_x_*, *q_y_*, and *q_z_*; (**p**–**s**) 3D trajectory comparisons of DPI-LSTM, PI-LSTM, D-LSTM, and LSTM with the ground truth.

Figure 10 shows that different architectures exhibit clear differences in trajectory reconstruction, position error evolution, and attitude variation. All four models are able to reconstruct the ground-truth trajectory to a reasonable extent. Although the baseline LSTM performs reasonably well in overall trajectory fitting, as reflected by the position–component curves and the 3D trajectory plots, its predictions in some regions rely more strongly on empirical data fitting and lack sufficient physical regularisation. This can also be observed from the locally enlarged position components in Figure 10d–f.

In contrast, the D-LSTM, which uses only the dual-branch structure, can alleviate task interference to some extent during the joint modelling of position and attitude. However, its improvements remain limited because it does not include explicit physical consistency constraints. The complete DPI-LSTM combines physical consistency constraints with a dual-branch decoupling mechanism, thereby establishing a clearer functional division between the attitude branch and the position branch.

**Figure 11:**
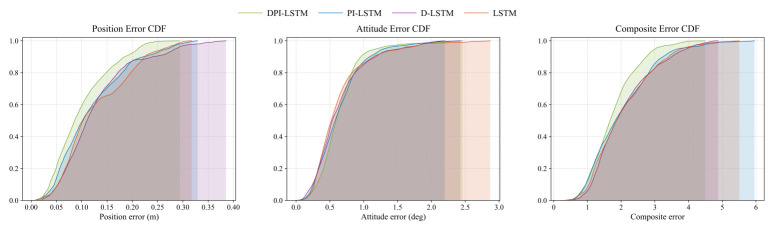
CDF comparison of position, attitude, and combined errors in the ablation experiments.

Figure 11 shows that the error distribution of DPI-LSTM is generally more concentrated, with its cumulative probability curve reaching higher probabilities at smaller error thresholds across most intervals. This suggests that its position and attitude estimation results exhibit better overall consistency and robustness. In the CDF comparison plots, the cumulative probability curves of all models show relatively similar trends for attitude error, with differences being less pronounced than those observed in the position error distribution. However, after introducing physical consistency constraints, the predicted attitude evolution becomes smoother and more consistent with the continuous characteristics of real motion.

The quantitative results of the ablation experiments further reveal the specific roles of the physical consistency constraints and the dual-branch design. As shown in Table 5, the complete DPI-LSTM achieves the best position estimation performance among the four architectures, with *MSE_pos_* = 0.00428 m^2^, *RMSE_pos_* = 0.0654 m, and *MAE_pos_* = 0.0484 m. This indicates that the joint introduction of the dual-branch structure and the physical consistency constraints contributes to improved position estimation accuracy under the tested conditions. In contrast, the baseline LSTM yields *MSE_pos_* = 0.00697 m^2^, *RMSE_pos_* = 0.0835 m, and *MAE_pos_* = 0.0605 m. While it still demonstrates good data-fitting capability, its overall performance remains inferior to that of DPI-LSTM.

Further comparison among the ablation variants shows that PI-LSTM improves the position metrics relative to the baseline LSTM: its *RMSE_pos_* decreases from 0.0835 to 0.0799, and its *MAE_pos_* decreases from 0.0605 to 0.0575. This indicates that explicitly introducing physical consistency constraints provides useful structured regularisation, helping the model learn predictions that are more consistent with classical inertial kinematic relationships and thereby improving position estimation performance. By contrast, the D-LSTM, which uses only the dual-branch structure, shows limited improvement in this set of experiments, with *RMSE_pos_* = 0.0843 m, which is comparable to that of the baseline LSTM. This indicates that, without the support of physical consistency constraints, structural decoupling alone is insufficient to consistently improve position estimation accuracy.

In terms of the attitude metrics, the differences among the four models are relatively small. DPI-LSTM achieves *MSE_att_* = 5.62 × 10^−5^, *RMSE_att_* = 0.00749, and *MAE_att_* = 0.00577, maintaining high overall accuracy and demonstrating good stability.

Overall, the ablation experiments indicate that the physical consistency constraints play an important role in improving position estimation accuracy, while the dual-branch architecture provides structural support for separately modelling position and attitude. The complete DPI-LSTM combines the dual-branch decoupling structure with physics-informed consistency constraints. The former separately models position and attitude through task-specific branches, whereas the latter establishes physical coupling between translational and rotational motion along the attitude–acceleration–velocity–position chain.

### 6.4. Cross-Sequence Evaluation

To further evaluate the model’s performance on non-overlapping sequences, this paper conducts a cross-sequence evaluation using an outdoor UZH-FPV sequence that is not used during training.

**Figure 12:**
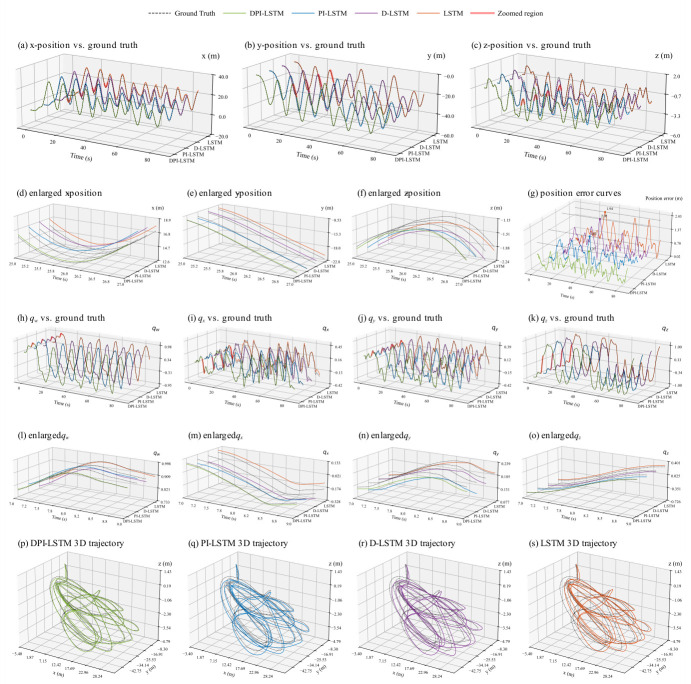
Comparison of position and attitude reconstruction and error curves in the cross-sequence evaluation: (**a**–**c**) position estimates along the x-, y-, and z-axes versus the ground truth; (**d**–**f**) enlarged views of the x-, y-, and z-position estimates; (**g**) position error curves of different methods; (**h**–**k**) quaternion estimates *q_w_*, *q_x_*, *q_y_*, and *q_z_* versus the ground truth; (**l**–**o**) enlarged views of the quaternion estimates *q_w_*, *q_x_*, *q_y_*, and *q_z_*; (**p**–**s**) 3D trajectory comparisons of DPI-LSTM, PI-LSTM, D-LSTM, and LSTM with the ground truth.

Figure 12 shows clear differences in position-and-attitude reconstruction performance among the compared models on the unseen UZH-FPV outdoor sequence. In the position–component curves, especially the locally enlarged views shown in Figure 12d–f, and in the attitude reconstruction curves shown in Figure 12h–o, the compared models exhibit different degrees of tracking deviation. Although the baseline LSTM, PI-LSTM, and D-LSTM are generally able to reconstruct the overall motion trend in their 3D trajectories, as shown in Figure 12q–s, they still exhibit noticeable deviations and error fluctuations during segments involving rapid local manoeuvres. Specifically, the baseline LSTM shows pronounced drift in both the position curves and the 3D trajectory.

The PI-LSTM shows improved overall trajectory fitting after the introduction of physical consistency constraints, indicating that these constraints can, to some extent, help regularise the learned motion patterns on the unseen sequence.

Meanwhile, the D-LSTM mitigates task interference in the joint learning of position and attitude through its dual-branch structure, but stability on the unseen sequence remains limited in the absence of explicit physical consistency constraints.

In contrast, DPI-LSTM achieves better agreement with the ground-truth values in terms of position components, attitude variations, and 3D trajectory reconstruction, as shown in Figure 12p. It also exhibits smoother position error evolution, as shown in Figure 12g. This indicates that the joint introduction of the dual-branch structure and physical consistency constraints improves the model’s estimation stability on the unseen sequence.

**Figure 13:**
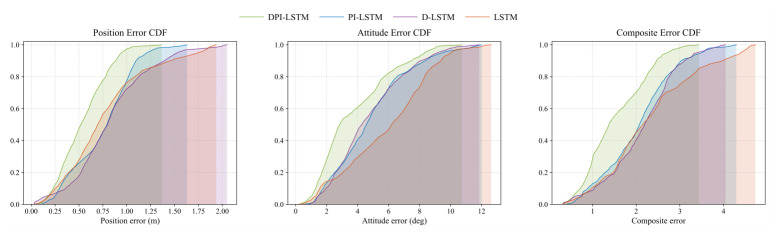
CDF comparison of position, attitude, and combined errors in the cross-sequence evaluation.

Figure 13 presents the CDF comparison of the position, attitude, and combined errors in the cross-sequence evaluation. DPI-LSTM reaches higher cumulative probabilities at smaller error thresholds over most intervals, indicating that its estimation errors are more concentrated than those of the other evaluated variants. This indicates that the joint introduction of the dual-branch structure and physical consistency constraints improves the model’s estimation stability on the unseen sequence.

Table 6 summarises the quantitative results on the unseen outdoor sequence. In terms of the position metrics, DPI-LSTM achieves the best performance among the four architectures, with *MSE_pos_* = 0.117 m^2^, *RMSE_pos_* = 0.342 m, *MAE_pos_* = 0.256 m, and *R*^2^*_pos_* = 0.995. Compared with LSTM, PI-LSTM, and D-LSTM, the *RMSEpos* of DPI-LSTM is reduced by approximately 33.2%, 26.8%, and 35.3%, respectively. This indicates that, under the sequence-level distribution shift within the UZH-FPV dataset, the combination of physical consistency constraints and the dual-branch structure improves the modelling of translational motion.

In terms of the attitude metrics, DPI-LSTM also achieved the best results, with *MSE_att_* = 0.00213, *RMSE_att_* = 0.0461, *MAE_att_* = 0.0353, and *R*^2^*_att_* = 0.953. Compared with LSTM, PI-LSTM, and D-LSTM, the *RMSE_att_* of DPI-LSTM is reduced by approximately 36.1%, 25.2%, and 24.5%, respectively. This indicates that, on the unseen outdoor sequence, the proposed method improves position estimation accuracy while maintaining stable attitude-estimation performance.

When the test sequence differs from the training sequences in scene and motion patterns, models that rely solely on data fitting are more prone to performance degradation. However, by introducing physical consistency constraints and a dual-branch structured modelling strategy, DPI-LSTM maintains more stable and accurate position and attitude estimation performance on the unseen sequence. Together with the same-distribution test results in Section 6.2, these cross-sequence results indicate that the proposed method maintains stable position and attitude estimation performance under both standard test conditions and moderate sequence-level distribution shifts.

To provide a preliminary evaluation of the computational efficiency of the proposed method, the structural complexity of DPI-LSTM was analysed. As the physical consistency losses are used only as regularisation losses during training, they do not introduce additional computational overhead during inference. Therefore, the inference complexity is mainly determined by the shared LSTM encoder and the two task-specific prediction branches. Table 7 summarises the number of trainable parameters, approximate model size, estimated computational cost, and inference latency for a single input window of 40 IMU samples.

### 6.5. Evaluation on the EuRoC Dataset

To further evaluate the effectiveness of the proposed dual-branch physics-informed structure under a different dataset distribution, an additional retraining experiment was conducted on the EuRoC Micro Aerial Vehicle (MAV) dataset [41]. The purpose of this experiment is to examine whether the proposed DPI-LSTM architecture remains effective when it is trained and evaluated on a different public UAV dataset with similar IMU-based position and attitude estimation data.

In this experiment, the easy sequences were used for training, the medium sequences were used for validation, and the difficult sequences were held out for testing. All four model variants, namely LSTM, PI-LSTM, D-LSTM, and DPI-LSTM, were trained under the same experimental setting. Importantly, no additional hyperparameter tuning was performed specifically for the EuRoC dataset. The same network configuration and training parameters were retained, including the input sequence length, LSTM hidden dimension, learning rate, and physical-loss weights. Therefore, the comparison focuses on whether the dual-branch architecture and physical consistency constraints remain beneficial in the target domain retraining setting. The retraining results are shown in Table 8.

As shown in Table 8, DPI-LSTM achieves the best overall performance among the four model variants after retraining on the EuRoC MAV dataset. For position estimation, DPI-LSTM obtains the lowest *MSE*, *RMSE*, and *MAE*, as well as the highest *R*^2^. For attitude estimation, DPI-LSTM also achieves the lowest *MSE*, *RMSE*, and *MAE*, together with the highest *R*^2^. Compared with PI-LSTM, which introduces physical consistency constraints without the dual-branch structure, DPI-LSTM reduces the *RMSE_pos_* from 0.111 to 0.108 and the *RMSE_att_* from 0.0856 to 0.0842.

These results indicate that the dual-branch structure and the physics-informed constraints provide structural benefits. Although the performance improvement is moderate, it is consistent across position, attitude, and overall position and attitude estimation metrics. As no EuRoC-specific hyperparameter adjustment was performed, the observed improvement suggests that the performance gain is more likely associated with the proposed dual-branch physics-informed structure rather than dataset-specific parameter tuning. This additional experiment further verifies that the proposed DPI-LSTM architecture remains effective after retraining on a different public UAV dataset.

## 7. Conclusions

This paper investigates an IMU-driven position and attitude estimation for quadrotor UAVs and conducts systematic experimental validation based on the UZH-FPV Drone Racing dataset. To address the susceptibility of traditional navigation methods under purely inertial conditions to noise, bias, and cumulative integration errors, a dual-branch physics-informed long short-term memory (DPI-LSTM) model is proposed. Experimental results demonstrate that the proposed method can effectively improve the stability and overall performance of position and attitude estimation under complex manoeuvring conditions.

The main conclusions are as follows:

1. To address the problem of error accumulation under purely inertial conditions, the proposed DPI-LSTM effectively suppresses the long-term drift associated with traditional inertial integration methods, achieving more stable and accurate position and attitude estimation, particularly in terms of suppressing long-term drift in position estimation under complex manoeuvres. These results indicate that the proposed method can reduce drift-like position estimation errors under the tested benchmark conditions.
2. In the joint regression task for position and attitude, the constructed dual-branch architecture achieves more stable overall estimation performance, indicating that this structure can mitigate task interference between position and attitude regression within the shared feature space and enhance representation capability across different motion modes.
3. Further ablation experiments demonstrate that introducing physical consistency constraints into a data-driven learning framework improves estimation stability and enhances cross-sequence performance within the UZH-FPV dataset. The additional EuRoC retraining results further confirm the structural effectiveness of the proposed DPI-LSTM, showing that the dual-branch physics-informed design can provide consistent improvements in position, attitude estimation under a new dataset setting.

Although the proposed DPI-LSTM achieves improved position and attitude estimation stability under the evaluated benchmark settings, several limitations should be acknowledged. The current framework remains a supervised learning method and relies on dataset-provided reference position and attitude annotations during training. The cross-sequence evaluation within the UZH-FPV dataset reflects sequence-level distribution shifts rather than full cross-domain generalisation, while the EuRoC experiment provides only preliminary external-dataset retraining validation. In addition, under IMU-only conditions, position and attitude estimation is still affected by sensor noise, bias, observability limitations, and long-duration drift accumulation. The physical consistency constraints serve as auxiliary regularisation and cannot completely eliminate these inherent limitations of inertial-only navigation. Although model complexity and inference latency have been analysed, embedded deployment, onboard energy consumption, and closed-loop UAV feedback validation remain to be investigated in future work.

From an engineering application perspective, the proposed method provides an effective data-driven implementation pathway for state mapping and dynamic modelling within UAV digital twin systems. Within the digital twin framework, continuous, stable, and accurate position and attitude estimation serves as a crucial foundation for realising core functions such as flight-status monitoring, trajectory prediction, and risk assessment. In addition, this capability is also valuable for precision agriculture, where reliable UAV position and attitude estimation can support the safety of low-altitude operations, improve the stability of crop monitoring and precision spraying tasks, and support reliable state mapping for agricultural UAV operations. By incorporating established inertial kinematic relationships into the deep temporal modelling process as loss constraints, DPI-LSTM improves position estimation stability under the evaluated benchmark conditions. This may reduce the dependence on external positioning devices under constrained sensing conditions and may improve the deployment flexibility of UAV digital twin systems in complex environments.

Future research will further explore online learning and multi-sensor fusion mechanisms and combine them with real-time communication and virtual–physical interaction technologies to advance the construction of UAV digital twin models oriented towards closed-loop updating and real-time feedback, thereby further enhancing the engineering value of the system in complex flight missions. In this future framework, the digital twin is expected to serve not only as a state-monitoring and prediction module but also as a supervisory layer for supporting closed-loop UAV control. At the same time, future work will investigate the deployment of the proposed framework in application-oriented scenarios such as precision agriculture, with the aim of supporting safer autonomous field operations and more reliable precision agriculture applications.

## Figures and Tables

**Table 1:** Main characteristics of the Snapdragon Flight IMU used in this study.

Item	Value/Description
IMU data source	Snapdragon Flight IMU stream
IMU sensor	InvenSense MPU-9250
Sampling frequency	500 Hz
Gyroscope noise	0.01°/s/√Hz; 0.1°/s-rms at 92 Hz DLPF
Gyroscope bias specification	Zero-rate tolerance: ±5°/s at 25 °C; temperature variation: ±30°/s
Accelerometer noise	300 μg/√Hz; 8 mg-rms at 94 Hz DLPF
Accelerometer bias specification	Zero-g tolerance: ±60 mg for X/Y axes; ±80 mg for Z axis

**Table 2:** Main implementation settings of DPI-LSTM.

Item	Value/Setting
Input window length	40 IMU samples
Temporal encoder	Two-layer LSTM
LSTM hidden dimension	128
Branch hidden dimension	64
Training stabilisation	Gradient clipping with maximum norm 0.5
Physical-loss weights	$\lambda_{quat}=0.1,\;$ $\lambda_{vec}=0.02,\;$ $\lambda_{acc}=5\times 10^{-3},\;$ $\lambda_{vel}=0.1,\;$ $\lambda_{pos}=0.1$

**Table 3:** Sample data from the UZH-FPV dataset.

	Timestamp	tx	ty	tz	qw	qx	qy	qz	ang_vel_x	ang_vel_y	ang_vel_z	lin_acc_x	lin_acc_y	lin_acc_z
1	0.4828	6.952171	3.424433	0.94715	0.4549	0.007125	0.006	0.890496	0.15766	0.10866	0.02024	2.365459	1.63763	4.989491
2	0.4848	6.95216	3.42438	0.94716	0.45485	0.007146	0.00596	0.890517	0.29827	0.08096	0.007457	3.08372	4.563325	13.12495
3	0.4868	6.952146	3.424329	0.94718	0.45482	0.007165	0.00593	0.890535	0.012783	0.128897	0.153398	2.537841	0.27773	11.15213
4	0.4888	6.952131	3.424279	0.94719	0.45479	0.007184	0.0059	0.89055	0.133158	0.035154	0.03835	0.019154	0.2873	12.03798
5	0.4908	6.952115	3.424231	0.94721	0.45476	0.007202	0.00588	0.890563	0.132093	0.08629	0.07457	0.105344	4.72613	9.03567
6	0.4928	6.952096	3.424184	0.94722	0.45474	0.00722	0.00587	0.890573	0.04261	0.10866	0.10759	1.08696	1.766912	7.872095
7	0.4948	6.952077	3.424138	0.94723	0.45473	0.007236	0.00586	0.890582	0.17151	0.01704	0.02876	0.541087	0.009577	8.834558
	……													
93	0.6648	6.950593	3.420943	0.94849	0.45444	0.007771	0.00602	0.890721	0.021305	0.01385	0.02024	0.45968	1.24019	11.14256
94	0.6668	6.950585	3.420912	0.9485	0.45445	0.007776	0.00602	0.890718	0.022371	0.075634	0.037284	2.250538	3.3806	9.840118
95	0.6688	6.950578	3.42088	0.9485	0.45446	0.007782	0.00603	0.890715	0.08735	0.02237	0	0.450108	1.426937	12.38275
96	0.6708	6.950571	3.420849	0.94851	0.45446	0.007789	0.00604	0.890712	0.06072	0.20666	0.013848	0.57939	1.0295	9.634218
97	0.6728	6.950565	3.420817	0.94852	0.45447	0.007795	0.00604	0.89071	0.01598	0.18003	0.017044	1.68551	2.83472	7.790692
98	0.6748	6.950558	3.420786	0.94853	0.45447	0.007801	0.00605	0.890707	0.03835	0.045806	0.014914	0.158017	2.64797	7.359738
99	0.6768	6.950552	3.420755	0.94854	0.45448	0.007808	0.00605	0.890705	0.024501	0.133158	0.0032	0.809236	0.22505	9.198476
100	0.6788	6.950546	3.420723	0.94855	0.45448	0.007814	0.00606	0.890702	0.02131	0.003196	0.04687	0.335187	1.39821	10.19446
	……													

The ellipses indicate omitted entries, and the complete dataset was used in the actual experiments rather than only the displayed rows.

**Table 4:** Quantitative comparison of different methods.

Method	MSE_pos_	RMSE_pos_	MAE_pos_	R^2^_pos_	MSE_att_	RMSE_att_	MAE_att_	R^2^_att_
Direct INS	519	22.7	12.9	−32.6	0.0592	0.243	0.0304	0.964
Bias INS	92.2	9.60	6.60	−274	0.0194	0.139	0.00960	0.992
Mahony INS	98.2	9.91	6.92	−273	0.0194	0.139	0.00991	0.992
RoNIN	0.00960	0.0980	0.0708	0.996	6.94 × 10^−5^	0.00833	0.00642	0.999
CNN–LSTM	0.00754	0.0869	0.0614	0.998	7.27 × 10^−5^	0.00852	0.00623	0.999
IONet	0.00691	0.0831	0.0600	0.997	4.98 × 10^−5^	0.00706	0.00555	0.999
DPI-LSTM	0.00428	0.0654	0.0484	0.998	5.62 × 10^−5^	0.00749	0.00577	0.998

**Table 5:** Quantitative results of the ablation experiments.

Method	MSE_pos_	RMSE_pos_	MAE_pos_	R^2^_pos_	MSE_att_	RMSE_att_	MAE_att_	R^2^_att_
LSTM	0.00697	0.0835	0.0605	0.997	6.03 × 10^−5^	0.00777	0.00570	0.998
PI-LSTM	0.00638	0.0799	0.0575	0.998	6.17 × 10^−5^	0.00786	0.00610	0.999
D-LSTM	0.00711	0.0843	0.0620	0.998	6.51 × 10^−5^	0.00807	0.00589	0.998
DPI-LSTM	0.00428	0.0654	0.0484	0.998	5.62 × 10^−5^	0.00749	0.00577	0.998

**Table 6:** Quantitative results of the cross-sequence evaluation.

Method	MSE_pos_	RMSE_pos_	MAE_pos_	R^2^_pos_	MSE_att_	RMSE_att_	MAE_att_	R^2^_att_
LSTM	0.263	0.512	0.349	0.995	0.00520	0.0721	0.0555	0.872
PI-LSTM	0.218	0.467	0.343	0.993	0.00380	0.0616	0.0491	0.914
D-LSTM	0.279	0.529	0.400	0.988	0.00373	0.0611	0.0495	0.926
DPI-LSTM	0.117	0.342	0.256	0.995	0.00213	0.0461	0.0353	0.953

**Table 7:** Computational complexity and inference latency of the evaluated model variants.

Method	Trainable Parameters	Model Size (FP32)	MACs/ Window	FLOPs/ Window	Latency (Batch = 1)	Latency (Batch = 256)
LSTM/PI-LSTM	256,455	0.978 MB	8.181 M	16.362 M	0.655 ± 0.060 ms	35.765 ± 1.136 ms
D-LSTM/DPI-LSTM	256,711	0.979 MB	8.181 M	16.363 M	0.608 ± 0.032 ms	35.756 ± 1.052 ms

**Table 8:** Target domain retraining results on EuRoC.

Method	MSE_pos_	RMSE_pos_	MAE_pos_	R^2^_pos_	MSE_att_	RMSE_att_	MAE_att_	R^2^_att_
LSTM	0.0123	0.111	0.0817	0.985	7.33 × 10^−3^	0.0856	0.0540	0.652
PI-LSTM	0.0123	0.111	0.0812	0.985	7.33 × 10^−3^	0.0856	0.0541	0.640
D-LSTM	0.0132	0.115	0.0819	0.984	7.47 × 10^−3^	0.0863	0.0548	0.649
DPI-LSTM	0.0116	0.108	0.0794	0.986	7.01 × 10^−3^	0.0842	0.0527	0.657

## Data Availability

The data analyzed in this study were derived from publicly available datasets. The UZH-FPV Drone Racing Dataset is available at https://fpv.ifi.uzh.ch/ (accessed on 13 November 2025) and https://fpv.ifi.uzh.ch/datasets/ (accessed on 13 November 2025); its accompanying publication is available with DOI: 10.1109/ICRA.2019.8793887. The EuRoC MAV Dataset is available at https://projects.asl.ethz.ch/datasets/euroc-mav/ (accessed on 7 June 2026) with DOI: 10.3929/ethz-b-000690084. No new datasets were generated in this study.

## Abbreviations

The following abbreviations are used in this manuscript:

UAV	Unmanned aerial vehicle
DT	Digital twin
UAVDT	Unmanned aerial vehicle digital twin
IMU	Inertial measurement unit
INS	Inertial navigation system
EKF	Extended Kalman filter
GNSS	Global navigation satellite system
LSTM	Long short-term memory
RNN	Recurrent neural network
CNN	Convolutional neural network
PINN	Physics-informed neural network
DPI-LSTM	Dual-branch physics-informed long short-term memory
PI-LSTM	Physics-informed long short-term memory
D-LSTM	Dual-branch long short-term memory
MSE	Mean squared error
RMSE	Root mean squared error
MAE	Mean absolute error
R^2^	Coefficient of determination
